# Association of Clubroot Resistance Locus *PbBa8.1* With a Linkage Drag of High Erucic Acid Content in the Seed of the European Turnip

**DOI:** 10.3389/fpls.2020.00810

**Published:** 2020-06-11

**Authors:** Zongxiang Zhan, Yingfen Jiang, Nadil Shah, Zhaoke Hou, Yuanwei Zhou, Bicheng Dun, Shisheng Li, Li Zhu, Zaiyun Li, Zhongyun Piao, Chunyu Zhang

**Affiliations:** ^1^College of Horticulture, Shenyang Agricultural University, Shenyang, China; ^2^National Key Laboratory of Crop Genetic Improvement, College of Plant Science and Technology, Huazhong Agricultural University, Wuhan, China; ^3^Institute of Crop Science, Anhui Academy of Agricultural Science, Hefei, China; ^4^Yichang Academy of Agricultural Science, Yichang, China; ^5^Collaborative Innovation Center for the Characteristic Resources Exploitation of Dabie Mountains, College of Biology and Agriculture Resource, Huanggang Normal University, Huanggang, China

**Keywords:** clubroot, *Brassica napus*, erucic acid, disease resistance, genetic improvement

## Abstract

Clubroot caused by *Plasmodiophora brassicae* is a severe threat to the production of *Brassica napus*, worldwide. The cultivation of resistant varieties is the most efficient and environmentally friendly way to limit disease spread. We developed a highly resistant *B. napus* line, ZHE226, containing the resistance locus *PbBa8.1*. However, ZHE226 seeds contain high erucic acid content, which limits its cultivation owing to its low edible oil quality. A segregation population of BC_3_F_2_ was developed by crossing ECD04, a resistant European turnip donor, with Huangshuang5, an elite variety with no erucic acid in its seeds, as a recurrent plant. Fine mapping using the bulk segregation analysis sequencing (BSA-Seq) approach detected *PbBa8.1* within a 2.9 MB region on chromosome A08. Interestingly, the previously reported resistance gene *Crr1a* was found in the same region. Genetic analysis revealed that the CAP-134 marker for *Crr1a* was closely linked with clubroot resistance (CR). Thus, *PbBa8.1* and *Crr1a* might be allelic for CR. Moreover, comparative and genetic analysis showed that high erucic acid in the seeds of ZHE226 was due to linkage drag of *fatty acid elongase 1* (*FAE1*) in the ECD04 line, which was located in the interval of *PbBa8.1* with a physical and genetic distance of 729 Kb and 1.86 cm, respectively. Finally, a clubroot-resistant line with a low erucic acid content was successfully developed through gene-specific molecular marker assistant selection from BC_4_F_4_. These results will accelerate CR breeding programs in *B. napus.*

## Introduction

Clubroot is caused by a soil-borne, biotrophic obligate pathogen, *Plasmodiophora brassicae* (Woronin) and is a severe disease of rapeseed (*Brassica napus*) and other cruciferous crop species worldwide (Dixon, 2009). The life cycle of clubroot pathogen is comprised of two stages: root hair infection stage and cortex infection stage. Gall formation in infected roots is the most characteristic symptom of the disease, causing obstruction in nutrient uptake and resulting in premature death of the plant (Johnson and Karling, 1944; Suwabe et al., 2003; Hwang et al., 2011). The resting spores of clubroot pathogen can survive in the soil for over 20 years, making it difficult to control infection with cultural or chemical measures (Voorrips, 1995). Clubroot severely impairs the sustainable and healthy development of the rapeseed industry, especially in China and Canada. Several strategies have been developed for controlling clubroot, among which breeding of resistant cultivars is considered to be the most economical and environmentally friendly method (Donald et al., 2001; Diederichsen et al., 2009; Shigeru et al., 2010). The majority of clubroot-resistant germplasm with resistance genes/quantitative trait loci (QTL) were derived from European fodder turnips and were successfully used in CR breeding programs in Chinese cabbage and canola (Diederichsen et al., 2006; Rahman et al., 2011). The first winter canola clubroot resistant line, cv. “Mendel,” originated from a resynthesized *B. napus* (AACC, 2*n* = 38) line and was developed through the crossing of *Brassica oleracea* “ECD15” (CC, 2*n* = 18) and *Brassica rapa* “ECD04” (AA, 2*n* = 20) (Diederichsen and Sacristan, 2010). “Mendel” was successfully crossed with the Canadian spring canola and several lines were developed with high resistance to a number of *P. brassicae* pathotypes in Canada (Rahman et al., 2014). To date, more than 13 resistance genes/QTLs have been mapped in five chromosomes of the A-genome of *B. rapa* (Huang et al., 2019). *Crr1* and *PbBa8.1* resistance genes are located in the same locus of the A08 chromosome (Chen et al., 2013). *Crr1* contains *Crr1a* and *Crr1b*, and *Crr1a* was successfully cloned by Katsunori et al. (2013).

The ZHE226 line (*B. napus*, AACC, 2*n* = 38), containing the resistance gene *PbBa8.1*, was derived by backcrossing with an elite rapeseed conventional variety, Huashuang5, while ECD04 was used as a CR donor plant (Zhan et al., 2015). ECD04 is an elite plant that contains 5 CR loci and is resistant to four different isolates of *P. brassicae* (Pb2, Pb4, Pb7, and Pb10) (Chen et al., 2013). The *PbBa8.1* gene provides resistance to a number of clubroot-causing field isolates. The improved CR homozygous line, ZHE226, containing this locus exhibited strong resistance to pathotype 4 of *P. brassicae* in severely infested fields in Anhui, Hubei, and Sichuan provinces of China. However, after several backcrosses with Huashuang5, ZHE226 seeds and their offspring developed an increased and unstable content of erucic acid (C22:1) ranging from 1.39 to 18.95% in different growing seasons, as measured using near-infrared ray (NIR) techniques, while the glucosinolate content in the seeds was similar to that of Huashuang5. Erucic acid is a long-chain monounsaturated fatty acid that accumulates in the seeds of cruciferous plants and reduces the quality of oil the plants produce. It is predominantly oxidized in peroxisomes rather than in mitochondria, and promotes the production of reactive oxygen species and various cytosolic lipid metabolites. In some cases, it causes cardiotoxicity in animals, such as rodents and pigs (Fumiaki et al., 2013). High levels of erucic acid ingested through cooking with rapeseed oil is associated with ocular and respiratory tract diseases (Vles et al., 1978; Wang et al., 2010). Intake of high levels of erucic acid may also affect fertility and prenatal development, and cause damage to the human myocardial fibers (Zhang et al., 2008). The maximum level of erucic acid is found in canola seeds where it makes up 5% of the total fatty acids present, and this is considered to be an acceptable amount (Singh et al., 2011; Tian et al., 2018). However, reducing the content of erucic acid in oil to an acceptable level, or close to negligible amounts, is one of the most significant breeding goals for improving the canola oil quality (Vesna et al., 2010). Therefore, selecting clubroot-resistant materials with low erucic acid content (CR-LEA) is crucial for rapeseed breeding programs.

The *fatty acid elongase 1* (*FAE1*) gene encodes a seed-specific enzyme of β-ketoacyl-CoA synthase that catalyzes the first condensation step in the elongation of very-long-chain fatty acids and limits erucic acid biosynthesis (Roscoe et al., 2001; Sun et al., 2013). The FAE1enzyme extends the fatty acid chain length from C18 to C20 and C22 (Lassner et al., 1996; Han et al., 2001). It was initially cloned in Arabidopsis by directed transposon tagging with the maize element activator (*Ac*) (James et al., 1995). Dorrell and Downey (1964) and Karim et al. (2016) indicated that erucic acid synthesis was determined by a single non-dominant gene, in *B. rapa* (*B*rassica *campestris*), whereas, Siebel and Pauls (1989) reported that erucic acid is simply inherited and controlled by two additive genes in *B. napus*. The two additive alleles are located on the A08 and C03 chromosomes in *B. napus*, accounting for ∼71% of the genetic variation in erucic acid synthesis (Qiu et al., 2006). The first oilseed cultivar with low erucic acid content (LEA) oilseed cultivar, Oro, was bred using the LEA Liho crop as parental material (Gang et al., 2008). The use of Oro as a donor plant has become a worldwide standard for the breeding of cultivars with LEA in seeds. Erucic acid content in ZHE226 was modestly high, although it was backcrossed with the LEA variety Huashuang5 for several generations. However, it is unclear whether *FAE1* is a linkage drag of *PbBa8.1*, considering both genes are located on the A08 chromosome (Fourmann et al., 1998; Chen et al., 2013). Aside from the high erucic acid content in the seeds, ZHE226 is a CR line with high seed yield performance, with a similar genetic background to the recurrent parental variety Huashuang5. Therefore, in this study, a relationship between the genetically close linkage of the CR locus *PbBa8.1* and the high erucic acid determining gene *FAE1* was revealed, and a valuable CR line with LEA content was developed. Overall, this study will accelerate CR breeding programs in *B. napus.*

## Materials and Methods

### Plant Materials

**FIGURE 1:**
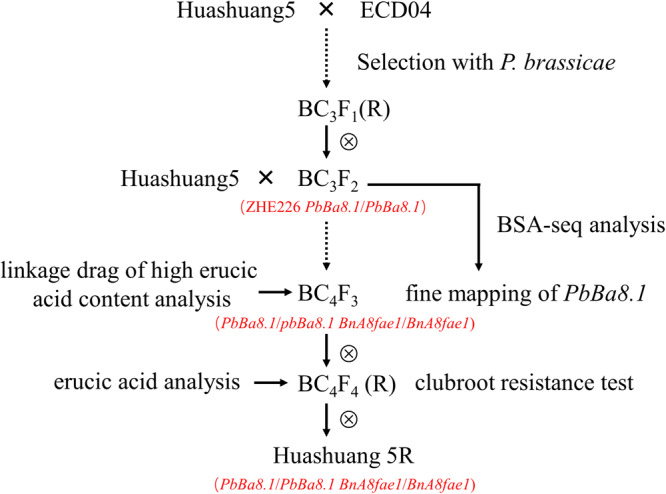
Flow chart of Huangshan5R clubroot-resistant material with very low erucic acid content.

The segregation population of BC_3_F_2_ with CR was developed by crossing/backcrossing of ECD04 (European turnip, donor plant) containing the CR gene *PbBa8.1* with Huangshuang5 (CR susceptible, recurrent plant), which is an elite conventional rapeseed variety with zero erucic acid and low glucosinolate content in the seeds. This population was used for bulked segregation analysis coupled with whole genome sequencing (BSA-Seq). Another segregation population, BC_4_F_3_, was developed by backcrossing BC_3_F_2_ homozygous for the *PbBa8.1* gene with Huashuang5 (Figure 1). Linkage analyses of *PbBa8.1* and *FAE1* were carried out for the generation of this line. European turnip ECD04, a CR donor plant containing *PbBa8.1*with a high concentration of erucic acid content in seed, was used as a control for *FAE1* gene cloning.

### *Plasmodiophora brassicae* Inoculation and Resistance Tests

*Plasmodiophora brassicae* samples with severe galling were collected from the Huangshan locality, amplified on *B. napus-*susceptible material, and stored at −20°C. The resistance test in the control condition was carried out as previously described by Chen et al. (2013). Briefly, frozen galls were thawed at room temperature, homogenized, and resting spores were extracted. The spore concentration was adjusted to10^7^ resting spores per milliliter in sterile distilled water and this spore suspension was used for host root inoculation. One-week-old seedlings were inoculated with resting spores by injecting 1 mL of the spore suspension into the root zone around the plant (Chen et al., 2013; Pang et al., 2018). The greenhouse day/night temperature was maintained at 25/20°C. The plants were watered regularly. Forty days post-inoculation (dpi), the plants were carefully dug out, their roots were washed, and roots were examined for resistance (Zhan et al., 2017).

### High-Throughput Sequencing

Genomic DNA from young leaves was extracted using the DNeasy Plant Kit (TIANGEN, Beijing, China). DNA quality and quantity were measured using a Nano-Drop 2000 (Thermo Fisher Scientific, Waltham, MA, United States). An equal amount of DNA (2.0 μg/sample) from 30 BC_3_F_2_-resistant plants were mixed to construct the resistant bulk sample (R-pool) and a similar amount of DNA from 30 BC_3_F_2_-susceptible plants with severe clubs were mixed to form susceptible bulk samples (S-pool). The DNA samples were sequenced using the Illumina HiSeq 2000 platform (Illumina, San Diego, CA, United States) with 20× read depth. Quality control (QC) parameters were set as follows: QC procedures were reads with ≥10% unidentified nucleotides, reads with >50% bases having phred quality <5, reads with >10 nt aligned to the adapter, and putative PCR duplicates generated by PCR amplification in the library construction process were removed, and ≤10% mismatches were allowed. The clean reads were mapped to the reference genome of *B. napus*^1^ using the Burrows-Wheeler Aligner (BWA).

### Gnome-Wide InDel Marker Development

SAMtools and Varscan^2^ were employed to screen the InDel markers (Koboldt et al., 2012). The flanking sequence of the InDel site was extracted for primer development using Primer3 software (Untergasser et al., 2012). The parameters were set as follows: ≤3 nucleotide mismatch for the primers were allowed, ≤2 gaps in the PCR production were allowed, and PCR production size was set to 50–250 base pairs (bp). All primers were screened with the template genomic sequences from *B. rapa* and *B. napus* by e-PCR. The markers with only one amplification product in *B. napus* that also anchored on the same chromosome of *B. rapa* were allowed.

### Bulked Segregation Analysis

SNP/InDel detection and variant annotation were performed for the two pools (R and S-pool) using the Unified Genotyper function (GATK software). The ANNOVAR tool was used to annotate SNPs or InDels based on the reference genome (Mckenna et al., 2010). The read depth information for homozygous SNPs in the offspring pools was obtained to calculate the SNP index (frequency). The SNP indices in both pools that were under 0.3 were filtered out. The sliding window method was employed to present the SNP index of the whole genome. The average SNP index in each window was used as the SNP index. Default settings included a window size of 100 kb at a step size of 20 kb. A 95% interval of the permutation test was selected as a candidate locus from the delta of all indices (Takagi et al., 2013).

### Molecular Cloning of the *BnaA8FAE1* Gene

Genomic DNA from young leaves of ZHE226, ECD04, and Huashuang5 was extracted using the CTAB method (Murray and Thompson, 1980). Primers were designed according to the gene sequence of *FAE1* (GU325717.1) published by NCBI^3^ The purified PCR products were amplified with Fast-Pfu Polymerase, ligated to the pEASY-Blunt vector (TransGen Biotech Co., Beijing, China) and transformed into *Escherichia coli* DH5α competent cells (Takara). At least four positive clones with a correct insertion size were selected and sequenced (AuGCT, Beijing, China). The gene-specific markers, AW and AM (Supplementary Table S1), were designed based on the SNP of *FAE1* that differed between Huashuang5 and ECD04 lines using the amplification refractory mutation system (ARMS) method (Newton et al., 1989).

### Erucic Acid Quantification in Seeds

To determine the erucic acid content and genotypes in the segregating BC_4_F_3_ population at the earliest stage, the half-seed technique was employed (Anand and Downey, 1981). Briefly, a portion of the seed (0.5 mg) was cut carefully without damaging the embryo and transferred to a 5 mL glass tube. Then, 1.5 mL of 2.5% methanol sulfate solution and 350 μL of methylbenzene was added. The glass tubes were carefully sealed and kept at 90°C for 30–45 min. After cooling to room temperature, 1 mL of double-distilled H_2_O (ddH_2_O) and 1 mL *n*-hexane were added to each tube, blended, and tubes were centrifuged at 1000 rpm for 5 min using a Heraeus Fresco 21 microcentrifuge (Thermo Fisher Scientific, Germany). The supernatant containing the fatty acid methylated ester was transferred to autosampler vials and 0.5 μL samples were injected and analyzed by gas chromatography (GC) (HP7890A, Agilent) at a nitrogen carrier gas flow rate of 30 mL/min. The initial oven temperature was 180°C for 2 min, followed by 10°C/min to a final temperature of 220°C, which was held for 12 min.

Fatty acid standards [palmitic acid (C16:0), stearic acid (C18:0), oleic acid (C18:1), linoleic acid (C18:2), linolenic acid (C18:3), eicosenoic acid (C20:1), and erucic acid (C22:1) were purchased from Sigma-Aldrich China (Shanghai)]. The mean values and standard deviations from three biological replicates were calculated. Quantification of each fatty acid was carried out by the percentage of peak values using corresponding standard samples (Zhang et al., 2013).

## Results

### Resistance Test and Fine Mapping of *PbBa8.1* in *B. napus*

**FIGURE 2:**
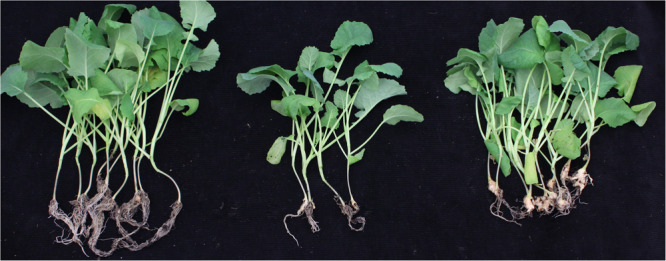
Clubroot resistant tests with pathogen of Huangshan in the BC_3_F_2_ generation. Root symptom evaluation of resistant BC_3_F_2_ individuals, susceptible individuals, and Huashuang5 (left to right).

A total of 933 BC_3_F_2_ individuals derived from ECD04 and Huashuang5 were inoculated with *P. brassicae* pathotype 4 collected from Huangshan, China. Of these individuals, 720 were resistant (R) to clubroot and 213 were susceptible (S), with a segregation ratio of 3:1, indicating a dominant pattern for inheritance of resistance [*N* = 933, χ^2^ = 2.49 < χ^2^0.05(1) = 3.84] (Figure 2). The BSA-resequencing method was employed for fine-mapping of the resistance locus. A total of 137,056,905 and 138,277,214 clean reads were obtained from R- and S-pools after filtering with the next generation sequencing (NGS) tool kit, respectively (Supplementary Table S2, SAR accession: PRJNA605484). Over 95% of the clean reads were mapped onto the genome of *B. napus* (*Brassica_napus*_v4.1.chromosomes) (Supplementary Table S2), and a total of 303,345 SNPs and 18,818 InDels were detected between the two pools.

**FIGURE 3:**
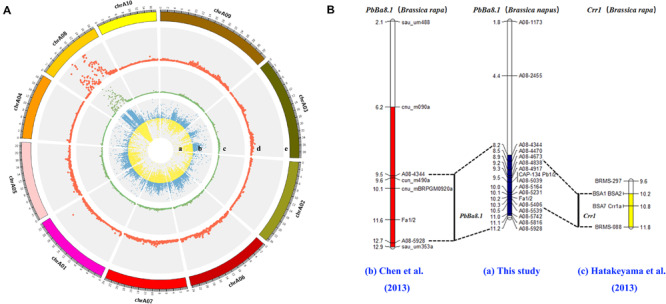
Fine mapping of *PbBa8.1* in *B. napus* and synteny analysis with other resistant loci. **(A)** SNP distribution in the A genome and candidate region prediction. **(B)** Physical mapping of *PbBa8.1* with InDel markers and synteny analysis with other resistant loci. The blue bar represents the candidate region of *PbBa8.1* in *B. napus*. The red bar represents the candidate region of *PbBa8.1* in *B. rapa*. The yellow bar represents the candidate region of *Crr1* in *B. rapa.*

The CR locus, *PbBa8.1*, was mapped to a physical region between 8.15 and 11.22 Mb on chromosome A08 of *B. napus* based on the ΔSNP index (Figure 3A). Approximately 57.32% of the SNPs were mapped to the A08 chromosome, and more than 12,000 SNPs were located in the candidate region. The large number of SNPs on the A08 chromosome may be due to the advanced population used in the experiment. A high-density physical map was then constructed (Figure 3A). A total of 121 indel markers were screened in the candidate region (Supplementary Table S3) with a density of 33.41 markers per Mb. Molecular markers were selected for synteny analysis, and the results revealed that the previously reported resistance gene, *Crr1*, overlapped with *PbBa8.1* on the A08 chromosome between the markers of BSA1 and BRMS-088 in the physical map of the present study, with an interval of 1.3 Mb (Figure 3B and Supplementary Table S1).

### Development of a Co-segregated Marker Linked to Clubroot Resistance Based on *Crr1a*

**FIGURE 4:**
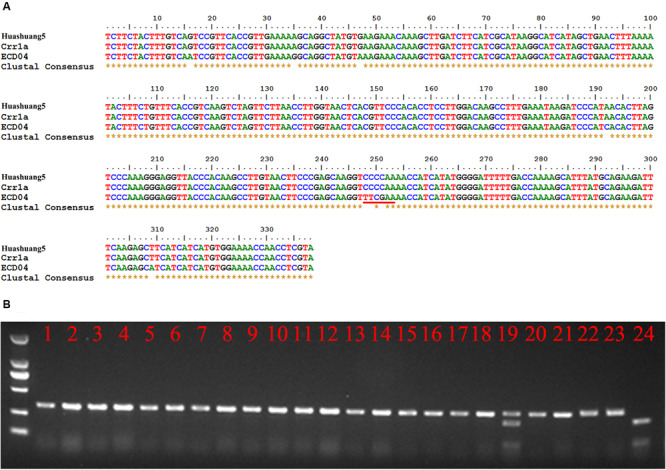
Gene-specific marker, CAP-134, designed based on sequence differences between Huashuang5 and ECD04. **(A)** Homologous nucleotide sequence alignment of *Crr1a* of ECD04 and Huashuang5. The red line indicates the restriction endonuclease sites of *Taq*I. **(B)** Genotype analysis of randomly selected susceptible individuals of the BC_3_F_2_ generation. Lines 1–22 show the susceptible plants. Lines 23 and 24 show Huangshuang5 and ECD04, respectively.

Although *Crr1a* was derived from “Siloga S2” and exhibits minor CR alone, while *PbBa8.1* is a dominant locus that is likely controlled by at least one of the divergent functional alleles of *Crr1a*. To prove this hypothesis, a pair of gene-specific primers were designed based on the *Crr1a* sequence and used to amplify a 380 bp fragment from the total genomic DNA of ECD04 and Huashuang5 as templates. Sequencing results revealed that the 380 bp sequence from Huashuang5 is exactly the same as the reported *Crr1a* wild type sequence, indicating this gene does not significantly impact CR in Huashuang5. Fortunately, variations were observed in the corresponding sequence of ECD04 (Figure 4A), and this region was sufficient to generate a cleaved amplified polymorphic sequences (CAPS) marker (CAP-134) based on the sequence differences (249–251 sequence position), resulting in an extra restriction enzyme cutting site for *Taq*I in the ECD04 genome (Figure 4A). Using the CAP-134 marker, 200 randomly selected lines from the S-pool of the above BC_3_F_2_ generation were genotyped and 196 were found to be homozygous for the marker, while four were heterozygous (Figure 4B). To confirm whether the above four heterozygous plants were infected by other mixed pathotypes in the field, the self-pollinated seeds from these four heterozygous individuals were planted in the same infested field as Huangshan5 in the next growing season. The results showed a 3:1 segregation ratio for resistance and susceptibility indicating that, rather than recombination, these four plants were randomly infected by other pathotypes in the field with mixed features. Further the co-segregation characteristics of CAP-134 with *PbBa8.1* was proven based on a large population.

### Cloning and Characterization of *FAE1*

**FIGURE 5:**
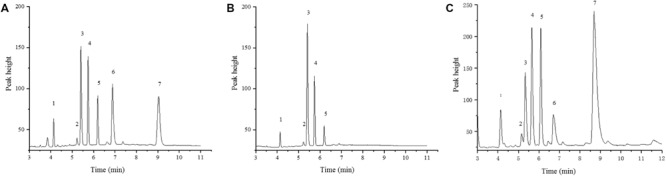
Gas Chromatograms of ZHE226, Huashuang5, and ECD04 seed fatty acid composition. **(A)** ZHE226; **(B)** Huashuang5; **(C)** ECD04. No. 1–7 represents palmitic acid (C16:0), stearic acid (C18:0), oleic acid (C18:1), linoleic acid (C18:2), linolenic acid (C18:3), eicosenoic acid (C20:1), and erucic acid (C22:1), respectively.

The FAE1 enzyme catalyzes the first condensation step in the elongation of very longchain monosaturated fatty acids (VLCMFA). There are two copies of *FAE1* on the C3 and A08 chromosomes in *B. napus* (*BnC3FAE1* and *BnA8FAE1*). The *FAE1* gene was mapped to chromosome A08 to a 10.18 MB region (*Brassica_napus*_v4.1.chromosomes), as shown in Figure 3B. The physical distance between CAP-134 and *BnA8FAE1*was approximately 729 Kb, indicating a linkage drag of the genes causing high erucic acid content and CR. In order to confirm this hypothesis, the oil composition of mature seeds from ZHE226, Huashuang5, and ECD04 were measured using GC. The erucic acid composition in ZHE226 and ECD04 seeds was 24.88 and 44.58%, respectively, (Supplementary Table S4). However, erucic acid was not detected in Huashuang5, which confirmed that the high erucic acid haplotype of *FAE1* in ZHE226 was inherited from ECD04 and not Huashuang5 (Figure 5). To further elucidate this result, genomic DNA from *BnA8FAE1* was isolated by PCR and then sequenced and aligned (Supplementary Figure S1). As expected, the sequences of ECD04 and ZHE226 were identical to GU325717.1 (*BnA8FAE1*); however, there was a single transition of cytosine (C) to thymine (T) at position of 845of the *FAE1* coding sequence in Huashuang5, which causes the serine (Ser) residue to transform to a phenylalanine (Phe) residue at amino acid position 282 (Table 1 and Supplementary Figure S1). These results indicate that this single C to T transition causes a loss of function mutation in *BnA8fae1* in Huashuang5.

**TABLE 1:** Sequence differences of *FAE1* between ECD04 and Huashuang 5.

**Origin**	**Number of nucleotide**	**Number of amino acid**	**Variation of nucleotide**	**Variation of amino acid**	**Gene function**
GU325717.1	1521	506	845 (C)	282 (Ser)	Y
ECD04	1521	506	845 (C)	282 (Ser)	Y
Huashuang 5R	1521	506	845 (C)	282 (Ser)	Y
Huashuang 5	1521	506	845 (T)	282 (Phe)	N

### Genetic Analysis of Clubroot-Resistant Loci *PBba8.1* and *BnA8FAE1*

**FIGURE 6:**
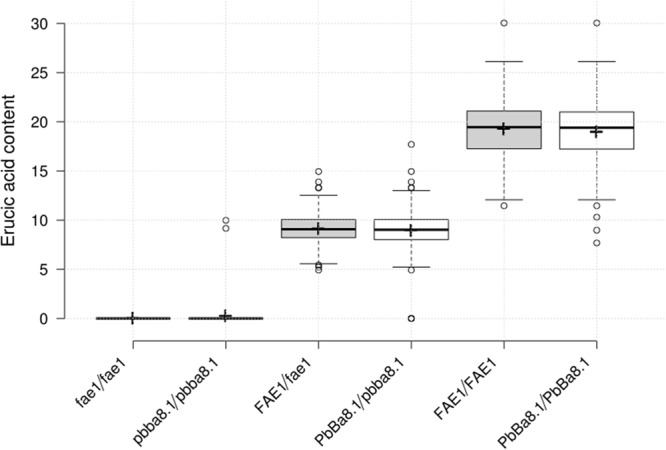
Erucic acid content of the different genotypes in the BC_4_F_3_ generation. Centerline represents the median; upper and lower boxes indicate upper and lower quartiles (Q3 and Q1) calculated with R software; the dotted line extends 1.5 times the upper and lower quartiles; circles indicate outliers; crosses represent sample means; *n* = 82, 80, 141, 141, 74, 75.

**FIGURE 7:**
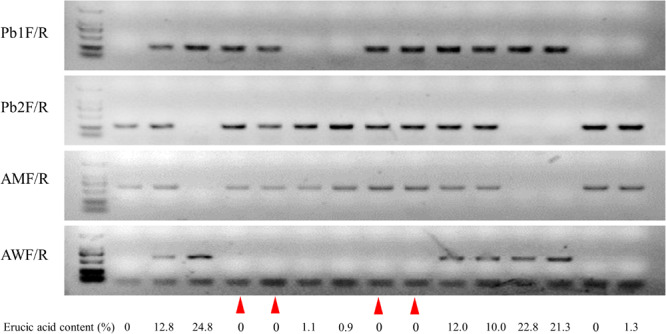
Genotypes and their corresponding erucic acid content in the BC_4_F_3_ generation. Lines 1–3 show Huashuang5, BC_3_F_1_ generation, and ZHE226, respectively. The remaining lines show heterozygous individuals selected from the BC_4_F_3_ generation. The red triangles represent the exchange of individuals with low erucic acid content.

In order to screen CR lines with LEA content, 296 individual seeds of the BC_4_F_3_ segregation population were used for erucic acid quantification, and genotyping of *BnA8FAE1/BnA8fae1* was performed using gene-specific marker pairs AW/AM. Phenotyping and genotyping results revealed that the average erucic acid content in the seeds of *BnA8fae1/BnA8fae1*, *BnA8FAE1/BnA8fae1*, and *BnA8FAE1/BnA8FAE1* were 0, 9.04, and 19.26%, respectively, indicating an additive effect of *BnA8FAE1* on erucic acid content. Using the gene-specific marker pair of Pb1/Pb2, converted from CAP-134 with ARMS method, the average erucic acid content in the seeds of *pbBa8.1/pbBa8.1*, *PbBa8.1/pbBa8.1* and*PbBa8.1/PbBa8.1* was 0.24, 8.83, and 18.95%, respectively (Figure 6). There were no significant differences in the average content of erucic acid between genotypes of *PbBa8.1/PbBa8.1* and *BnA8FAE1/BnA8FAE1*, *BnA8FAE1/BnA8fae1* and *PbBa8.1/pbBa8.1*, and *BnA8fae1/BnA8fae1* and *pbBa8.1/pbBa8.1*. This confirmed that *BnA8FAE1* is the linkage drag of *PbBa8.1*, and that *FAE1* derived from ECD04 has an additive effect on erucic acid accumulation. A total of 11 individuals with recombination occurring between the two loci were identified and 4 of them were heterozygous for the *PbBa8.1* locus with zero erucic acid in the seeds (Supplementary Table S5), because they carried the *BnA8fae1*gene. The genetic distance between *PbBa8.1* and *BnA8FAE1* was calculated to be approximately 1.86 cm based on the recombination rate in *B. napus* (Figure 7).

### Breeding for CR-Inbred Lines Without High Erucic Acid Content

**FIGURE 8:**
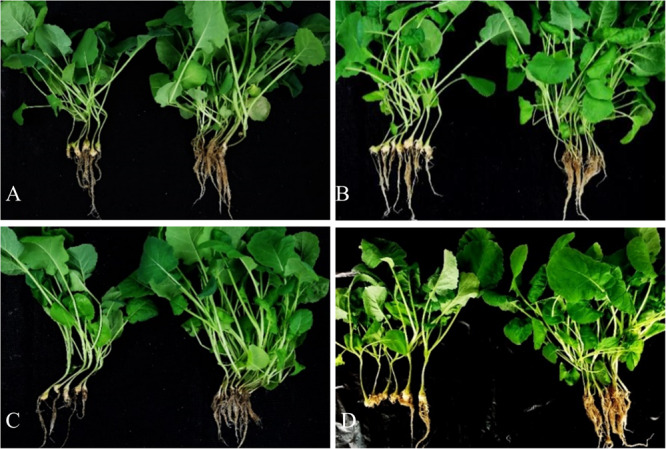
Clubroot resistance of four independent CR-LEA line. Panels **(A–D)** show Huashuang5 and the others show homozygous disease-resistant lines without erucic acid.

The above four recombinant lines (Supplementary Table S4) heterozygous for *PbBa8.1* as confirmed by Pb1/Pb2,with no erucic acid content as confirmed by GC analysis, were selected and self-pollinated to obtain homozygous CR lines. To confirm the erucic acid content of the self-pollinated seeds of these four recombinant lines, the NIRS technique was used to measure fatty acid composition. Very LEA contents of 2.8, 1.0, 1.0, and 1.4%were found in each of the four lines. Subsequently, the four different self-pollinated populations were tested for CR in the Huangshan region. A total of 173 homozygous and stable CR individuals were obtained by associated marker selection of the CR lines. Further, 16 randomly selected self-pollinated homozygous CR plants from the four different BC_4_F_4_ populations were and selected for quality analysis by NIRS. The erucic acid content of all these plants was less than 1.5% (Supplementary Table S6). Additionally, four lines were randomly selected for CR tests in the greenhouse. In contrast to the control plants, these lines exhibited durable resistance to the clubroot pathogen collected at the Huangshan locality (Figure 8).

## Discussion

Clubroot is a serious threat to rapeseed production globally, and particularly in China. Breeding of highly resistant varieties is the most effective way to control this disease (Diederichsen et al., 2009). CR turnips are widely used for CR breeding and resistance gene mapping. Most CR loci are race-specific. Previously, we developed a CR line, ZHE226, that contains the *PbBa8.1* gene, which confers strong resistance against *P. brassicae* pathotype 4 (Zhan et al., 2015). In the early generation of this elite line, the erucic acid content was not determined because it was expected that the offspring should have characteristic LEA and glucosinolate contents. However, in the BC_3_F_2_ generation, a high erucic acid content in the seed oil was observed in all CR lines, although glucosinolate content was the same as that of the parental Huangshuang5 line. In the present study, we predicted that the *PbBa8.1* locus might be closely linked with the *FAE1* gene that is located in the same chromosome, and could increase erucic acid levels. Fine mapping revealed that a linkage drag of high erucic acid determined by the haplotype *BnA8FAE1* is associated with the CR locus *PbBa8.1*. The recombination rate between the candidate genes, *PbBa8.1* and *BnA8FAE1*, was approximately 1.86%. In this study, a novel line, Huashuang 5R, with a very low seed erucic acid content and high resistance to clubroot was developed successfully. *PbBa8.1* exhibited dominant resistance against pathogen type 4 collected from the Huangshan region (Anhui Province). These results are consistent with the fact that *PbBa8.1* revealed a high resistance against a number of *P. brassicae* field isolates from Anhui, Sichuan, and Hubei provinces (Shah et al., 2019).

Our fine-mapping data revealed that *Crr1a* and *PbBa8.1* might be allelic and present in the same region in the genetic linkage map. However, *Crr1a* was cloned from the resistant line G004, encoding a Toll-Interleukin-1 receptor/nucleotide-binding site/leucine-rich repeat (TIR-NB-LRR) protein. Genetic analysis of the F_2_ population showed that the heterozygous *Crr1* locus demonstrates a minor resistance against Ano-01 (Katsunori et al., 2013). Together with *Crr2*, *Crr1* synergistically enhanced resistance (Suwabe et al., 2003).Thus, we can conclude that *PbBa8.1* and *Crr1* may be allelic, but differ in function. However, further study is required to explore whether other resistance genes are present in this region.

Interspecific hybridization breeding is aimed at introgression of a desirable trait from a wild donor line into an elite receptor line. However, undesirable and desirable traits are sometimes closely linked to each other in the alien segment from the donor that integrates into the genome of the receptor; this phenomenon is called linkage drag. Linkage drag can be removed by backcrossing. The probability of linkage drag depends on homology and the distance between two loci; the closer the linkage is, the more difficult it is to separate it, as it may require a large population and many generations of backcrossing. For example, linkage drag between high glucosinolate content in seeds and the restoration of Ogr-INGA cytoplasmic male sterility from the radish into *B. napus* took more than 10 years to relieve (Primard-Brisset et al., 2005). In tomato, the linkage breakdown of nematode resistance and undesirable fruit characteristics took 12 years to separate (Acquaah, 2012). However, in rice blast resistance, linkage drag was effectively removed in two cycles of recombinant selection (Feng et al., 2019). Cao et al. (2010) reported that backcross breeding and marker-assisted selection are effective tools to break linkage drag between low oil content and erucic acid in canola cultivars. Interestingly, in the current study, the linkage drag between the CR resistance locus and high erucic acid was relieved in 3 years, indicating a significantly high homology in the region of *PbBa8.1* of the turnip ECD04 to that of *B. napus* Huashuang5. Linkage drag could have been disrupted earlier if a large segregation population was used in the early segregated generations.

The partial allelic sequence results showed that *Crr1a* is identical to Huashuang5 (Figure 4). However, no genes homologous to *Crr1a* were predicted after Blast against the whole genome of *B. napus*. This is reasonable since Huashuang5 is completely susceptible to *P*. *brassicae*. Further, the marker, CAP-134, derived from the different sequences between ECD04 and Huashuang5, co-segregated with CR and could be very useful for promoting CR breeding for canola.

Minor galls were observed (approximately 8%) in heterozygous lines harboring *PbBa8.1*. However, a 3:1 segregation ratio for CR was repeatedly obtained when self-pollinated plants from heterozygous lines were tested in the field or in greenhouse conditions. On the other hand, the mixture of the clubroot pathogens with variance in virulence and uneven distribution in the field could be the cause of the discrepancy. Similar results were reported by Strelkov et al. (2016). Therefore, we suggest that successful fine mapping of CR genes, single spore isolation, and genetic recombinant studies should be employed in future programs. It is also crucial that the desired lines carefully self-pollinate first and then be examined for inoculation tests for the next generation.

## Data Availability Statement

All datasets generated for this study are included in the article/Supplementary Material.

## Author Contributions

ZZ analyzed the data, performed the experiments, and drafted the manuscript. YJ performed the experiments and helped to draft the manuscript. NS helped to analyze the data. ZH and BD contributed to perform the experiments. SL and LZ helped to analyze the data. ZL and ZP helped to draft the manuscript. CZ conceived the study, participated in its coordination, and helped to draft the manuscript. All authors read and approved the final manuscript.

## Conflict of Interest

The authors declare that the research was conducted in the absence of any commercial or financial relationships that could be construed as a potential conflict of interest.

## Footnotes

1 http://www.genoscope.cns.fr/brassicanapus/

2 http://varscan.sourceforge.net

3 https://www.ncbi.nlm.nih.gov

## Supplementary Material

FIGURE S1 Nucleotide sequence alignment of FAE1 between ECD04 and Huasuang 5. Red frame indicated the Nucleotide mutation site.

TABLE S1 All the primer sequences used in this study.

TABLE S2 The statistics of the R- and S-pools sequence alignment to the reference genome.

TABLE S3 List of the InDel markers designed in the candidate region.

TABLE S4 Fatty acid composition in seeds of ZHE226, Huashuang5 and ECD04.

TABLE S5 Recombinant plants and their erucic acid content detected in BC_3_F_2_ generation.

TABLE S6 Seed oil quality detection of 16 CR-LEA lines selected in BC_3_F_4_ generation and Huashuang5.
