# Supplementary figures and images for: Association of Clubroot Resistance Locus PbBa8.1 With a Linkage Drag of High Erucic Acid Content in the Seed of the European Turnip
Source: Front Plant Sci. 2020 Jun 11;11:810. doi: 10.3389/fpls.2020.00810 (PMC7301908; doi:10.3389/fpls.2020.00810)

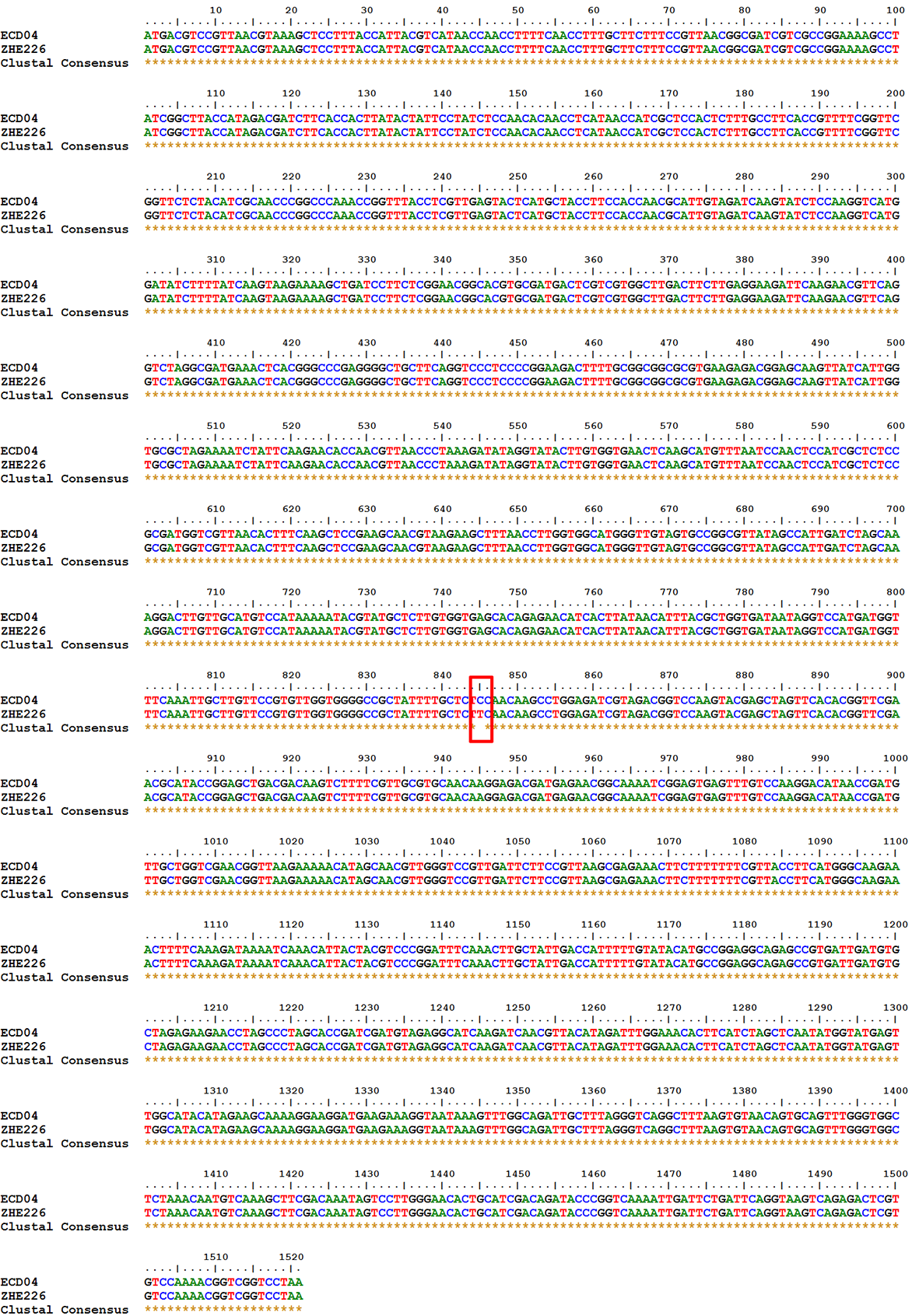

Supplement: FIGURE S1 — Nucleotide sequence alignment of FAE1 between ECD04 and Huasuang 5. Red frame indicated the Nucleotide mutation site. [file Image_1.tif]
