# Supplementary material for: Association of Clubroot Resistance Locus PbBa8.1 With a Linkage Drag of High Erucic Acid Content in the Seed of the European Turnip
Source: Front Plant Sci. 2020 Jun 11;11:810. doi: 10.3389/fpls.2020.00810 (PMC7301908; doi:10.3389/fpls.2020.00810)
Supplement: TABLE S2 — The statistics of the R- and S-pools sequence alignment to the reference genome. [file Table_2.doc]

**Association of clubroot resistance locus *PbBa8.1* with a linkage drag of high erucic acid content in the seed of the European turnip**

**Zongxiang Zhan^1ξ^, Yingfen Jiang^2,3ξ^, Nadil Shah^2^, Zhaoke Hou^2^, Yuanwei Zhou4, Bicheng Dun^2^, Shisheng Li^5^, Li Zhu^5^, Zaiyun Li^2^, Zhongyun Piao^1*^, Chunyu Zhang^2*^**

1-College of Horticulture, Shenyang Agricultural University, Shenyang 110866, Liaoning, China

2-National Key Laboratory of Crop Genetic Improvement and College of Plant Science and Technology, Huazhong Agricultural University, Wuhan430070, China

3-Institute of Crop Science, Anhui Academy of Agricultural Science, Hefei 230001, Anhui, China

4-Yichang Academy of AgriculturalScience, Yichang443004, Hubei, China

5-Collaborative Innovation Center for the Characteristic Resources Exploitation of Dabie Mountains and College of Biology and Agriculture Resource, Huanggang Normal University, Huanggang, Hubei, China

ξThese author contributed equally to this work.

*****Corresponding authors: Chunyu Zhang, [zhchy@mail.hzau.edu.cn](mailto:zhchy@mail.hzau.edu.cn)

ZhongyunPiao, [zypiao@syau.edu.cn](mailto:zypiao@syau.edu.cn)

Table S2 The statistics of the R- and S-pools sequence alignment to the reference genome

| Sample | Total_reads | Duplicates(%) | Unmapped(%) | Mapped(%) |
| --- | --- | --- | --- | --- |
| S-pool | 137056905 | 13740992(10.026%) | 5613529(4.096%) | 131443376(95.904%) |
| R-pool | 138277214 | 15050292(10.884%) | 5791575(4.188%) | 132485639(95.812%) |
