# Supplementary material for: Association of Clubroot Resistance Locus PbBa8.1 With a Linkage Drag of High Erucic Acid Content in the Seed of the European Turnip
Source: Front Plant Sci. 2020 Jun 11;11:810. doi: 10.3389/fpls.2020.00810 (PMC7301908; doi:10.3389/fpls.2020.00810)
Supplement: TABLE S3 — List of the InDel markers designed in the candidate region. [file Table_3.doc]

**Association of clubroot resistance locus *PbBa8.1* with a linkage drag of high erucic acid content in the seed of the European turnip**

**Zongxiang Zhan^1ξ^, Yingfen Jiang^2,3ξ^, Nadil Shah^2^, Zhaoke Hou^2^, Yuanwei Zhou4, Bicheng Dun^2^, Shisheng Li^5^, Li Zhu^5^, Zaiyun Li^2^, Zhongyun Piao^1*^, Chunyu Zhang^2*^**

1-College of Horticulture, Shenyang Agricultural University, Shenyang 110866, Liaoning, China

2-National Key Laboratory of Crop Genetic Improvement and College of Plant Science and Technology, Huazhong Agricultural University, Wuhan430070, China

3-Institute of Crop Science, Anhui Academy of Agricultural Science, Hefei 230001, Anhui, China

4-Yichang Academy of AgriculturalScience, Yichang443004, Hubei, China

5-Collaborative Innovation Center for the Characteristic Resources Exploitation of Dabie Mountains and College of Biology and Agriculture Resource, Huanggang Normal University, Huanggang, Hubei, China

ξThese author contributed equally to this work.

*****Corresponding authors: Chunyu Zhang, [zhchy@mail.hzau.edu.cn](mailto:zhchy@mail.hzau.edu.cn)

ZhongyunPiao, [zypiao@syau.edu.cn](mailto:zypiao@syau.edu.cn)

**Table S3 List of the InDel markers designed in the candidate region**

| **ID** | **start site of PCR in chromosome** | **ending site of PCR in chromosome** | **Forward primer (5'-3')** | **Reverse Primer (5'-3')** | **Products size (bp) of ECD04** | **Products size (bp) of *B.napus*** | **InDel size** |
| --- | --- | --- | --- | --- | --- | --- | --- |
| chrA08 | 8198795 | 8198942 | GCCATTGCTTGACATTGTTG | TCATTGTATGCATTGCTCCTTT | 144 | 148 | -4 |
| chrA08 | 8199759 | 8199854 | TCGTCGGTGTGAGAGAGAGA | TTGACCGAGACAATGGTGTG | 114 | 96 | 18 |
| chrA08 | 8207506 | 8207598 | GTGCCTGAAGCAGATAAGGC | AGATGAGAGCTTGTCGCCAT | 98 | 93 | 5 |
| chrA08 | 8207752 | 8207892 | TTCGATGAATCCTCCTCGTC | GCTTCACCTTCACCTTCACC | 147 | 141 | 6 |
| chrA08 | 8209360 | 8209457 | TCCACTATCTGAATTTGGGGA | GTGTGGGCTTCTTTTCTTGG | 101 | 98 | 3 |
| chrA08 | 8337723 | 8337853 | TCTAAAACCAAAATTAAATTGAAGAGA | GGTTTGCTTTCCTGTTCGTT | 128 | 131 | -3 |
| chrA08 | 8338007 | 8338139 | TGTTGTTACCACGGTGCAAT | ACCTTTGGTGGTAGTGGTGG | 136 | 133 | 3 |
| chrA08 | 8338740 | 8338852 | CAAGCAAAATTCCGACACAA | GAACAGGAGGAACAGGTGGA | 117 | 113 | 4 |
| chrA08 | 8339307 | 8339446 | CTGTTGTTGCTGCTGGTGAT | TGGGCTCCTACAACCGTAAC | 146 | 140 | 6 |
| chrA08 | 8345627 | 8345771 | CGGCTCACTGTCAGGAATCT | TGGACTTCTGCTGACATACCTT | 150 | 145 | 5 |
| chrA08 | 8540914 | 8541018 | CCCAAACCGTAAGAACTTGG | TGACGTGAAGGCTGTTGAGT | 113 | 105 | 8 |
| chrA08 | 8599357 | 8599479 | TGTTGCTAAGCTCCATCACG | TGATGTACACAGTCTCGCCA | 137 | 123 | 14 |
| chrA08 | 8662847 | 8662977 | CGTCAAGAGATTACTGGTCTTCC | CACACAAAAATCACTGTAGGCAA | 127 | 131 | -4 |
| chrA08 | 8764304 | 8764446 | ATTGCTCTCGCCAATCTGTT | GAGCAGTCAGCATTTGTCCA | 149 | 143 | 6 |
| chrA08 | 8766847 | 8766955 | AAAAATGTCAAATCGACACTCC | GCATGCATGTATCTAGTGAAGCA | 114 | 109 | 5 |
| chrA08 | 8780710 | 8780841 | TTGGCATTGGAGTAAAAAGTGA | GAGAGCAGAACCCATTTCACA | 128 | 132 | -4 |
| chrA08 | 8782180 | 8782318 | GAAACAACAATTGGTTGCCA | TCTTCTTTCTGGGCTTAGCG | 149 | 139 | 10 |
| chrA08 | 8790877 | 8791019 | GGCAAAGCGTAACTCTCAGG | ATCCAGGTGACGCTATCGAA | 150 | 143 | 7 |
| chrA08 | 8875664 | 8875801 | TGAAGTATCCCAACGACATCA | GGAAGCAATAGGTGAGTGAGG | 148 | 138 | 10 |
| chrA08 | 8910312 | 8910457 | CGGTGATGCACAAATTACCA | CAGCACCTGTGAACATAAACA | 142 | 146 | -4 |
| chrA08 | 8919917 | 8920044 | CATCGAGACCTGATTAAAACCC | CTTCGCTCCGTTGTAGTCGT | 131 | 128 | 3 |
| chrA08 | 9161153 | 9161317 | CCAAATGGAGCTCTTCCTCA | GGCTTGTGTTGGGTCATCAT | 143 | 165 | -22 |
| chrA08 | 9164921 | 9165084 | TTGTTTTCGATCGTGTATGTCT | CCACTGTCCTGTTGAAACAAAG | 150 | 164 | -14 |
| chrA08 | 9165494 | 9165614 | TGAACATGGCTACAAACGTGA | ATTCGCTTCCACTGCCATT | 115 | 121 | -6 |
| chrA08 | 9165893 | 9166025 | TCTGCCATGCAAAGGAATTA | AAGTCTCTATCTACATCTCCAGTCCT | 140 | 133 | 7 |
| chrA08 | 9181811 | 9181956 | AAAATAAAAATTCAGAACAATAGAAGC | TGCCGCATGAAATTAAGACTC | 142 | 146 | -4 |
| chrA08 | 9181811 | 9181956 | AAAATAAAAATTCAGAACAATAGAAGC | TGCCGCATGAAATTAAGACTC | 142 | 146 | -4 |
| chrA08 | 9186120 | 9186220 | ATATAGATGTGTCAAGTGGTTTTAGG | CTTTAAGTTCTCAATCACTCATAAACA | 93 | 101 | -8 |
| chrA08 | 9183120 | 9183257 | TGTGTTCAAGGTTTGTACTTCACTG | TCTGAGCTTTAAAAGATATCAAACGA | 146 | 138 | 8 |
| chrA08 | 9192907 | 9193042 | CAGCAACAAAACCTTGTGCC | TGTGCCTCTGTGAGTCGTTC | 133 | 136 | -3 |
| chrA08 | 9193021 | 9193132 | GTGAACGACTCACAGAGGCA | GCGAGTGGGTCCTACTGATG | 116 | 112 | 4 |
| chrA08 | 9193113 | 9193220 | CATCAGTAGGACCCACTCGC | AAGAAGAGCGATGACAACTGTG | 103 | 108 | -5 |
| chrA08 | 9194021 | 9194166 | CAAAGGCAGAGAGATTCACCA | CGGACTACATGAGGCTGTCA | 150 | 146 | 4 |
| chrA08 | 9198408 | 9198502 | AAAGAACGTGTATGCCCTGC | TGCCTCCTTCAAAAACATCC | 85 | 95 | -10 |
| chrA08 | 9198408 | 9198502 | AAAGAACGTGTATGCCCTGC | TGCCTCCTTCAAAAACATCC | 85 | 95 | -10 |
| chrA08 | 9198408 | 9198502 | AAAGAACGTGTATGCCCTGC | TGCCTCCTTCAAAAACATCC | 85 | 95 | -10 |
| chrA08 | 9200466 | 9200573 | TGAAAATTGTGCATTTCTCTGG | TGGTGACGATAACTGCTTGC | 104 | 108 | -4 |
| chrA08 | 9205472 | 9205590 | GTGAGGTCCGTTGTAGCCAT | CTCACGGAAACGCCTCTAGT | 104 | 119 | -15 |
| chrA08 | 9235051 | 9235199 | TGGTGGTTCGCTAGTTATCAGA | AGTTCATTGCAGACCACCAA | 145 | 149 | -4 |
| chrA08 | 9294067 | 9294195 | TGCCAGAAGCTTTGTACCCT | TCGTTGTACTGCACAATGGC | 121 | 129 | -8 |
| chrA08 | 9296273 | 9296418 | CGATTCGAAATCACGATTGA | CTTCTTCCTTCGTCGTCGTC | 138 | 146 | -8 |
| chrA08 | 9296273 | 9296419 | CGATTCGAAATCACGATTGA | TCTTCTTCCTTCGTCGTCGT | 139 | 147 | -8 |
| chrA08 | 9310134 | 9310287 | GCCATCAATGCGGTTTAAGT | CCTTTTCACGGATTACCCAA | 150 | 154 | -4 |
| chrA08 | 9381317 | 9381428 | CACCTTGAGAACACGAAGAGC | CTTATTTGCTGCACCTCTGC | 122 | 112 | 10 |
| chrA08 | 9393125 | 9393261 | TGTGCTAAAAAGGCCATTGA | TGAACCACTTACAGTAAAAACGAAA | 133 | 137 | -4 |
| chrA08 | 9393125 | 9393261 | TGTGCTAAAAAGGCCATTGA | TGAACCACTTACAGTAAAAACGAAA | 133 | 137 | -4 |
| chrA08 | 9393983 | 9394132 | TGCAACGAGGTAATGTCACG | CACATCCCACAAGTAACCGA | 147 | 150 | -3 |
| chrA08 | 9429439 | 9429555 | AGCAACAGGAGGAGGTGAGA | TGATAAAACATGTAATTGTGATTTGC | 121 | 117 | 4 |
| chrA08 | 9453391 | 9453522 | TGCCATCTGGAGATGAGTTG | GAATACAAAATCATGCGCGA | 126 | 132 | -6 |
| chrA08 | 9509351 | 9509475 | TAACTTGCCATCATCCCCTC | TGCTCAAAATCTTGTGGCTG | 118 | 125 | -7 |
| chrA08 | 9509652 | 9509796 | CTCAGCAAAGCACCATCAAA | TCCAGCTGATGATGTCAAGG | 141 | 145 | -4 |
| chrA08 | 9509652 | 9509796 | CTCAGCAAAGCACCATCAAA | TCCAGCTGATGATGTCAAGG | 141 | 145 | -4 |
| chrA08 | 9509652 | 9509796 | CTCAGCAAAGCACCATCAAA | TCCAGCTGATGATGTCAAGG | 141 | 145 | -4 |
| chrA08 | 9523852 | 9523975 | CCGATCGGATCATACCCTAA | GGAAAAGAAAGGTGCGAGTG | 130 | 124 | 6 |
| chrA08 | 9597175 | 9597319 | TTATTGACGGATGGCAAATG | GGGTTTTGTTCAGTGGTCACA | 149 | 145 | 4 |
| chrA08 | 9666037 | 9666161 | TTGGCGTTTTCTTTGTCAGA | ATCCTGATGGACTTTTTGCG | 120 | 125 | -5 |
| chrA08 | 9697373 | 9697514 | TCTTAGCCAACCCTCGACAC | ATCTCTTCGGGGTTTCGTTT | 132 | 142 | -10 |
| chrA08 | 9832217 | 9832356 | CTGCAATTCGCAACTAACCA | GGTCAACAGAAAATGGCCTG | 131 | 140 | -9 |
| chrA08 | 10011783 | 10011888 | TGGATTCTCGTTCTTGCCTT | CCATTGGGGCTGTTTGATAG | 109 | 106 | 3 |
| chrA08 | 10028058 | 10028130 | TCATCTCAAGTATCTATCTCATCAGTG | ATTGCTTCCTGACACTGCGT | 79 | 73 | 6 |
| chrA08 | 10031511 | 10031645 | CAGCCTACTCTGTTTTAACTTCTGC | CCATGTTACAGTGGGCTTCA | 128 | 135 | -7 |
| chrA08 | 10040981 | 10041098 | CGACCATACTGCCATACGTG | GGCCTACCTCAGAACCTTCA | 115 | 118 | -3 |
| chrA08 | 10049370 | 10049518 | TGTTGATTTAGGAGGTCAGTTTTG | CGGCCCATTATTTTCCACA | 146 | 149 | -3 |
| chrA08 | 10050678 | 10050768 | TCTACCACAGTTGGCATTCA | TCGATTAACTTGCAACTCAAGAA | 98 | 91 | 7 |
| chrA08 | 10051065 | 10051219 | CCAAACTCTGATGCTCCCCT | CCGTAGACACCCTTATTGGG | 150 | 155 | -5 |
| chrA08 | 10056844 | 10056979 | GAAAGGTGGAGGACGGTACA | GCCGAGAAAATGAAATCAAAA | 133 | 136 | -3 |
| chrA08 | 10064048 | 10064158 | TTGAATGGTGATTGTCGTCC | TCAACTGTGCTGCGGTTTTA | 121 | 111 | 10 |
| chrA08 | 10077243 | 10077365 | TGTGTAGCAAGCAGAGCACA | CAGTTCAGAGAAAATACAATTGGC | 119 | 123 | -4 |
| chrA08 | 10077342 | 10077480 | GCCAATTGTATTTTCTCTGAACTG | AGAAACACAGCCTGGCATTT | 149 | 139 | 10 |
| chrA08 | 10080852 | 10081001 | CACACGAAAGCAGTAAAGAACC | TCGCAGATGTGATGATCTGA | 145 | 150 | -5 |
| chrA08 | 10121581 | 10121738 | TTGTTGGAACGACCAAAAGA | CAAAGTGATGGACAAACCTGA | 150 | 158 | -8 |
| chrA08 | 10138415 | 10138538 | TCAACGAATTCAACAACCCA | ATCGCCACATTTTCTACCGA | 120 | 124 | -4 |
| chrA08 | 10141971 | 10142083 | GTGCTTTGGTTTTTGTGGCT | CAATGCTTTTTGTTTGGCTTT | 116 | 113 | 3 |
| chrA08 | 10151894 | 10152007 | TCGTAGATGTGACGGTGGAA | TGTCATGCTCCTACGGCTAA | 117 | 114 | 3 |
| chrA08 | 10152174 | 10152270 | TGCAAGATCAAGCAATGTGA | TTTCTTGGATCATGTAACGCA | 93 | 97 | -4 |
| chrA08 | 10153014 | 10153139 | TGGTTATCTCCGGTTTTGCT | TCATCAACAGCATGACTGACC | 132 | 126 | 6 |
| chrA08 | 10156645 | 10156767 | GTCGGCCTACAAAACGAAAA | CACATGTGCGTTTCTAAACACTT | 126 | 123 | 3 |
| chrA08 | 10183755 | 10183865 | CACTCTCCGTCGTCTTCTCC | CCGGCTTCTTCTCCAACTTT | 102 | 111 | -9 |
| chrA08 | 10198628 | 10198744 | GGAAAACATATTGCTTTTTCGG | CCACATGATCAGCGTCTTTG | 120 | 117 | 3 |
| chrA08 | 10284980 | 10285104 | GGTCTTAACCGTTTTTGCCA | TGGGCGCTACAGTTTAATCC | 115 | 125 | -10 |
| chrA08 | 10305331 | 10305482 | GGGACAGGTTAGTGAATGCC | TTAAGATCGACCCATCAGGC | 142 | 152 | -10 |
| chrA08 | 10316138 | 10316235 | ATAAACGATGGAACCACGCA | GCGAGCTTGCAGATTTTCTT | 93 | 98 | -5 |
| chrA08 | 10489744 | 10489916 | TATCCATATCCGGGCTGTGT | CCCCTCCTACCATGATTCCT | 131 | 173 | -42 |
| chrA08 | 10530214 | 10530348 | TGTTCTGATGTGATTGTACAGAGG | TGAAACCCGCATTTAAGAAGA | 143 | 135 | 8 |
| chrA08 | 10530214 | 10530348 | TGTTCTGATGTGATTGTACAGAGG | TGAAACCCGCATTTAAGAAGA | 143 | 135 | 8 |
| chrA08 | 10364576 | 10364720 | TCAGATATCTTCGGATCGGG | ACCCGAATTTGCGAACTAAA | 148 | 145 | 3 |
| chrA08 | 10364576 | 10364720 | TCAGATATCTTCGGATCGGG | ACCCGAATTTGCGAACTAAA | 148 | 145 | 3 |
| chrA08 | 10389422 | 10389553 | AAGACGATTTTGACATGTAACGAC | CCGAGAAAGAATACTAAAATTGTGC | 126 | 132 | -6 |
| chrA08 | 10389422 | 10389553 | AAGACGATTTTGACATGTAACGAC | CCGAGAAAGAATACTAAAATTGTGC | 126 | 132 | -6 |
| chrA08 | 10480591 | 10480691 | GAGATGAGCTGGGCAAACAT | ACACGTCCACCCTCTATTGC | 119 | 101 | 18 |
| chrA08 | 10480722 | 10480851 | GGCAATGAAGGACCAGTTGT | CCCGTGAACGTGGAGTAAAT | 127 | 130 | -3 |
| chrA08 | 10619451 | 10619577 | TTGTTTTCTGTAGACTTGGCCT | CCACACAAGTTATGTTGCTGTTC | 119 | 127 | -8 |
| chrA08 | 10702667 | 10702784 | CGTATTTGGAGCTTACCGGA | TGTTCTTCAGCTGTTTCTGCT | 126 | 118 | 8 |
| chrA08 | 10739437 | 10739573 | ATAGTACCAAGCCCTCACGG | TTTGAACATCATTTGGCTGA | 132 | 137 | -5 |
| chrA08 | 10742992 | 10743113 | CATCTGAAACCTCCTGATGATG | AGGGTTGCAAATGTTGTGAA | 147 | 122 | 25 |
| chrA08 | 10767315 | 10767425 | CCTTATACTAAGCGACGCGG | ATGGAGTTCAATACGACGGC | 117 | 111 | 6 |
| chrA08 | 10841578 | 10841722 | AAACACGTGAGCGAGCTTCT | GCTTCAAGCCATGTCCTCTC | 148 | 145 | 3 |
| chrA08 | 10885586 | 10885674 | CTGTTCTTGGAACCTGGAGC | CAGGTGGTGTGAGTGACGAC | 86 | 89 | -3 |
| chrA08 | 10891058 | 10891198 | CCGTGAGACGTAACGAATGA | GGACTTGTCCACAGGGCTT | 147 | 141 | 6 |
| chrA08 | 10914507 | 10914640 | AGCAGGTTCACTCCCATCAG | AAGGGAATGATTTTTGCTGG | 137 | 134 | 3 |
| chrA08 | 10952406 | 10952535 | TGAGGATGTGGATGCTTTGA | CCAAGGTACGTGACTGCAAA | 127 | 130 | -3 |
| chrA08 | 10961447 | 10961571 | CTTCAAGAACGATGGGGAAC | TTCTTACCATTTGCAGCGAC | 134 | 125 | 9 |
| chrA08 | 11038011 | 11038128 | ACTCGAAGAAGCTCACAGCC | ATTCCGGTTGGTTCTGTTTG | 122 | 118 | 4 |
| chrA08 | 11038011 | 11038128 | ACTCGAAGAAGCTCACAGCC | ATTCCGGTTGGTTCTGTTTG | 122 | 118 | 4 |
| chrA08 | 11043253 | 11043394 | TCCTAGCTTCGTTTCACGGT | TGATTTTTGTCGGTAACTAAATGA | 146 | 142 | 4 |
| chrA08 | 11043516 | 11043666 | ACGAAGAAAGGACAGAGCGA | TTGAGCAAGCACGAGATCAG | 124 | 151 | -27 |
| chrA08 | 11043638 | 11043779 | CTCGATCTGCTGATCTCGTG | CCTAAAACGACGTCGGAAAA | 150 | 142 | 8 |
| chrA08 | 11043638 | 11043779 | CTCGATCTGCTGATCTCGTG | CCTAAAACGACGTCGGAAAA | 150 | 142 | 8 |
| chrA08 | 11046886 | 11046998 | TGTTCACACCTACAAAAATGAGG | TTTGGTAGTTGTTGTTGTCACG | 119 | 113 | 6 |
| chrA08 | 11047470 | 11047593 | CCATATCTTGAGGAGCCGTG | AGGAAACAAAAACGACCAACA | 149 | 124 | 25 |
| chrA08 | 11107400 | 11107551 | TCAAAACCAAAGGAAATCAAGG | TTACCGTTTTGATCCCCAAA | 137 | 152 | -15 |
| chrA08 | 11107406 | 11107551 | CCAAAGGAAATCAAGGGGAT | TTACCGTTTTGATCCCCAAA | 131 | 146 | -15 |
| chrA08 | 11121906 | 11122000 | TGAATGCTTCAGTGACAGGC | GATGAGGGACGTTGCATCTAA | 101 | 95 | 6 |
| chrA08 | 11122839 | 11122992 | CATCAAGCAACCACCCAATA | TCCAAGGCATATGGTTAGTTCA | 143 | 154 | -11 |
| chrA08 | 11138886 | 11139004 | GCGAGTTGGAGAGCGAATAG | TGCTTCGGAATCGTTACACTT | 133 | 119 | 14 |
| chrA08 | 11149904 | 11150042 | GGGAAGCGACGACTACTGAG | GGGTGATCGATCGAGAGAGA | 130 | 139 | -9 |
| chrA08 | 11164566 | 11164711 | GCAACTGAGGCTATTGCTGG | CAGTTCCTGCAAAATGGGTA | 149 | 146 | 3 |
| chrA08 | 11173551 | 11173673 | GGGAAGGTCTGTTCCCAACT | GCACAGCAGAAGAAGCAATG | 131 | 123 | 8 |
| chrA08 | 11173654 | 11173762 | CATTGCTTCTTCTGCTGTGC | GCCAGTCCAGATGTGATCCT | 112 | 109 | 3 |
| chrA08 | 11221049 | 11221170 | GTTCCGGTCTCAGTAGTCGC | GTCACAGACTCCCCGTCATT | 125 | 122 | 3 |
| chrA08 | 11227981 | 11228103 | TGTGAATTGGCTTGGTTTGA | TCCTCCTGAACTTCCCGTAA | 120 | 123 | -3 |
